# Augmented Reality in Physical Therapy: Systematic Review and Meta-analysis

**DOI:** 10.2196/30985

**Published:** 2021-12-15

**Authors:** Maria Jesus Vinolo Gil, Gloria Gonzalez-Medina, David Lucena-Anton, Veronica Perez-Cabezas, María Del Carmen Ruiz-Molinero, Rocío Martín-Valero

**Affiliations:** 1 Department of Nursing and Physical Therapy University of Cadiz Cadiz Spain; 2 Clinical Management Unit Rehabilitation Intercentre-Interlevel University Hospitals of Puerto Real and Cadiz Cadiz Bay-La Janda Health District Cadiz Spain; 3 Institute for Biomedical Research and Innovation of Cádiz Cadiz Spain; 4 Department of Physical Therapy University of Malaga Malaga Spain

**Keywords:** augmented reality, physical therapy, rehabilitation, functionality

## Abstract

**Background:**

Augmented reality (AR) is a rapidly expanding technology; it comprises the generation of new images from digital information in the real physical environment of a person, which simulates an environment where the artificial and real are mixed. The use of AR in physiotherapy has shown benefits in certain areas of patient health. However, these benefits have not been studied as a whole.

**Objective:**

This study aims to ascertain the current scientific evidence on AR therapy as a complement to physiotherapy and to determine the areas in which it has been used the most and which variables and methods have been most effective.

**Methods:**

A systematic review registered in PROSPERO (International Prospective Register of Systematic Reviews) was conducted following PRISMA (Preferred Reporting Items for Systematic Reviews and Meta‐Analyses) recommendations. The search was conducted from July to August 2021 in the PubMed, PEDro, Web of Science, Scopus, and Cochrane Library scientific databases using the keywords *augmented reality*, *physiotherapy*, *physical therapy*, *exercise therapy*, *rehabilitation*, *physical medicine*, *fitness*, and *occupational therapy*. The methodological quality was evaluated using the PEDro scale and the Scottish Intercollegiate Guidelines Network scale to determine the degree of recommendation. The Cochrane Collaboration tool was used to evaluate the risk of bias.

**Results:**

In total, 11 articles were included in the systematic review. Of the 11 articles, 4 (36%) contributed information to the meta-analysis. Overall, 64% (7/11) obtained a good level of evidence, and most had a B degree of recommendation of evidence. A total of 308 participants were analyzed. Favorable results were found for the Berg Balance Scale (standardized mean change 0.473, 95% CI −0.0877 to 1.0338; *z*=1.65; *P*=.10) and the Timed Up and Go test (standardized mean change −1.211, 95% CI −3.2005 to 0.7768; *z*=−1.194; *P*=.23).

**Conclusions:**

AR, in combination with conventional therapy, has been used for the treatment of balance and fall prevention in geriatrics, lower and upper limb functionality in stroke, pain in phantom pain syndrome, and turning in place in patients with Parkinson disease with freezing of gait. AR is effective for the improvement of balance; however, given the small size of the samples and the high heterogeneity of the studies, the results were not conclusive. Future studies using larger sample sizes and with greater homogeneity in terms of the devices used and the frequency and intensity of the interventions are needed.

**Trial Registration:**

PROSPERO International Prospective Register of Systematic Reviews CRD42020180766; https://www.crd.york.ac.uk/prospero/display_record.php?RecordID=180766

## Introduction

### Background

New technologies are rapidly emerging in our society to streamline, optimize, and perfect some of the activities we perform in our day-to-day lives [1]. Among them is augmented reality (AR), which comprises generating new images from digital information in the real physical environment of a person, simulating an environment where the artificial and real are mixed [2]. AR must be differentiated from virtual reality (VR), in which additional data such as sound, text, or video are introduced, giving rise to multimedia virtual environments. AR is derived from VR but blends these virtual environments with real ones, enhancing the interaction with real life [3].

AR is currently being used in different fields such as advertising [4], psychology [5], medicine [6,7], and physiotherapy [8]. In physiotherapy, it has been developed mainly for motor and cognitive rehabilitation, which is considered a new method of intervention. AR can be used as a working tool and to complement the treatment conducted by the physiotherapist, as it generates safe environments that are similar to the patient’s real environment [9]. Rehabilitation using AR has shown better results than repetitive movements practiced alone as AR allows better orientation of the exercises toward objectives with greater patient motivation and is enjoyable to use [10].

AR technologies have significant advantages: they provide new experiences to patients during physiotherapy sessions, increasing engagement and improving physical outcomes [11]; they can create interesting opportunities to provide low-cost physiotherapy at home [12,13]; and the physiotherapist can perform and evaluate different outcomes using these tools with data analysis [14]. Although lack of technological maturity and access to devices are their weaknesses [15], various types of interfaces are emerging to ensure user interaction with the AR rehabilitation environment, including wearable smart sensors, sensors embedded in the environment, and mobile devices that improve accessibility to this type of technology [16]. Despite these possible benefits, there are few studies on AR used in physiotherapy, unlike VR, which has been studied in more pathologies, mostly of the neurological type, such as stroke [17-19], cerebral palsy [20], multiple sclerosis [21,22], Parkinson disease [23,24], spinal cord injury [25,26], and chronic pain [27].

Of the few investigations that have been conducted on AR, most were performed on healthy people with the aim of determining strategies that could later be used in the clinic [28]. Interest in studying AR has also grown in certain areas, such as the kinematic analysis of gait parameters [28,29], the functionality of the upper limb [30,31], or the early diagnosis of breast cancer–related lymphedema [32]. Positive results on balance and mobility have also been achieved when using dance with AR devices, with high adherence [33]. However, no improvement was found in the use of AR for the performance of daily living tasks in patients with Alzheimer disease [4].

Recently, a protocol for interactive AR-based telerehabilitation in patients with adhesive capsulitis was published [34] and another was published about people with hereditary spastic paraplegia, in which gait adaptability training was treated with a treadmill equipped with AR [35]; however, their results have not yet been published. In 2010, a review was conducted [36] in which most of the AR studies analyzed were in the prototype development phase and not yet ready for general practice, although they did show promising results.

### Objective

In the given context and taking into account all the advantages that the use of this kind of tool could have in physiotherapy, this review aims to determine how progress has been made in this regard, with the objective of ascertaining the current scientific evidence on AR therapy as a complement to physiotherapy, determining in which areas it has been used the most and which variables and methods have been most effective.

## Methods

A systematic review and meta-analysis was conducted and registered in PROSPERO (International Prospective Register of Systematic Reviews; CRD42020180766) using the PRISMA (Preferred Reporting Items for Systematic Reviews and Meta‐Analyses) guidelines [37].

### Search Strategy

A search of scientific evidence published from 2011 to August 2021 was conducted between July and August 2021 in the following scientific databases: PubMed, PEDro, Web of Science, Scopus, and Cochrane Library. In addition, gray literature (the TESEO database of doctoral theses in Spain, OpenGrey, and Grey Literature Database) and AR conference proceedings were searched. The keywords *augmented reality*, *physiotherapy*, *physical therapy*, *exercise therapy*, *rehabilitation*, *physical medicine*, *fitness*, and *occupational therapy* were used, combining them by means of the Boolean operators AND and OR in the different searches in English or Spanish.

### Criteria for Considering Studies

The criterion that was taken into account for selecting the articles was clinical trials published in indexed scientific databases. The selected intervention was AR used with patients aged >18 years with some pathology of the musculoskeletal system of neurological or physical origin that was subsidiary to improvement in any physical measure analyzed in an objective and standardized way. Duplicate studies, qualitative trials, case reports, single-subject studies, reviews, meta-analyses, studies conducted on healthy individuals, and studies using VR were excluded.

### Study Selection and Data Extraction Process

After performing the search, potentially relevant articles were identified after reading the title and abstract and eliminating duplicates. All studies identified in the searches were assessed for inclusion by 2 independent reviewers (MJVG and GGM). Any disagreements were resolved through discussions to reach a consensus. The following information was extracted from each included article: authors, year of publication, study population, type of intervention, number of participants, mean age, frequency of sessions per week, time of each session, total duration of the intervention, outcome measures, measurement instrument, and results obtained.

### Assessment of the Methodological Quality

To assess the quality of the trials used for the review, we used the PEDro scale [38], which comprises 11 items related to the domains of selection, performance, attribution bases, and information. A study with a PEDro score of ≥6 was considered as evidence level 1 (6-8: good and 9-10: excellent), and a study with a score of ≤5 was considered as evidence level 2 (4-5: acceptable and <4: poor) [39]. The recommendation grades of the different studies were presented using the Scottish Intercollegiate Guidelines Network scale [40].

### Risk of Bias Analysis

The risk of bias was calculated for each study using the Cochrane Collaboration tool [41], referring to the following types of bias: selection bias, performance bias, detection bias, attrition bias, reporting bias, and other bias. The risk of bias and study quality were calculated by 2 reviewers. In cases of doubt, the final decision was determined through discussion by including a third reviewer.

### Statistical Analysis

The effect size measure was the difference in the standardized mean change with raw standardization (SMCR) between the intervention (AR) and control groups [42,43] for 2 dependent end points: standardized mean change of the Berg Balance Scale (BBS) and the Timed Up and Go (TUG) test, with improvement after treatment indicated by positive values in the BBS and negative values in the TUG. The difference in SMCR was estimated in such a way that a greater difference in the intervention group was indicated by positive values in the BBS and negative values in the TUG. Standardized mean differences, sampling variances, and covariances were estimated according to Gleser and Olkin [44]. A multivariate random effects model with restricted maximum likelihood estimation was used, allowing for a different effect depending on the outcome and adding random effects to each outcome within each study. The goodness of fit was evaluated using sensitivity analyses [45] and likelihood profile plots. Publication bias was evaluated using contour-enhanced funnel plots [46]. The analyses were performed using the metafor package (GNU General Public License Version 2) [47] of the R software (R Foundation for Statistical Computing) [48].

## Results

### Selection of Studies

**Figure 1:**
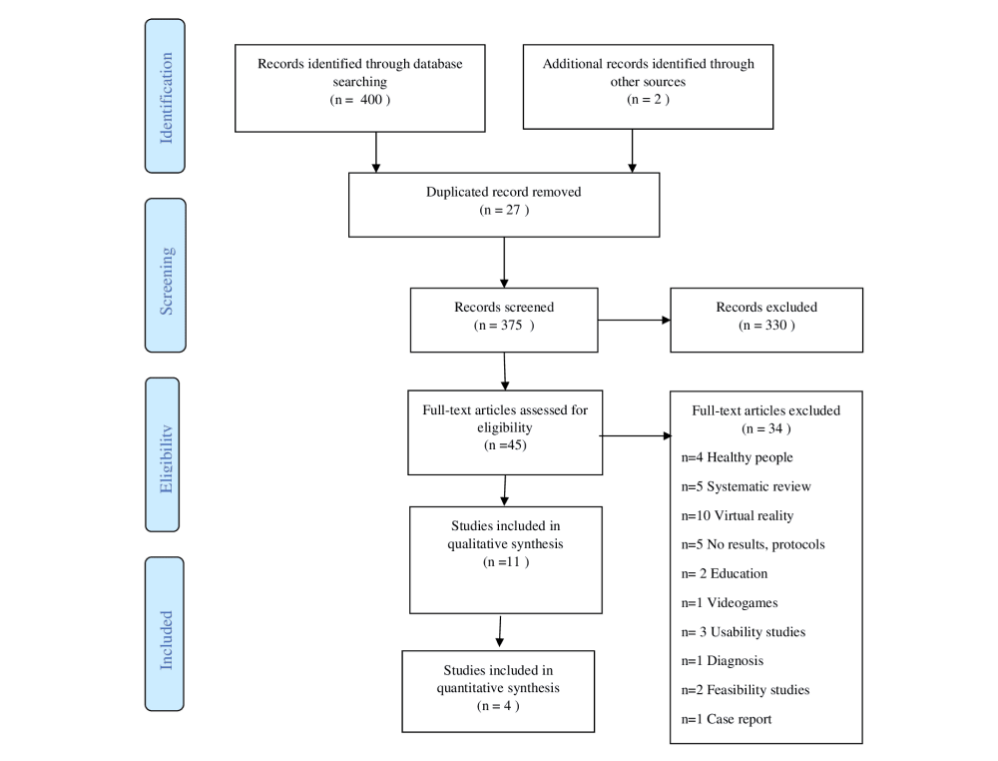
Flow diagram. Graphical representation of the process of search and selection of studies.

The entire selection process during the corresponding phases is detailed in Figure 1.

### Evaluation Outcomes

The sample size was variable, with the largest sample (75 patients) being in the study by Rothgangel et al [49] and the smallest (10 people) being in the study by Jung et al [50]. The included studies contained information on a total of 308 patients, of whom 89 (28.9%) had a stroke [50-52], 89 (28.9%) had amputations [49,53], 114 (37%) were geriatric patients [54-57], and 16 (5.2%) had Parkinson disease [58]. In terms of the age of the participants, the highest average was 76.4 in the study by Lee et al [55], and the lowest was 47.4 in the study by Kim et al [52]. It should be noted that of the 11 studies, 6 (55%) analyzed the effects of AR on the lower limb [49,50,52,54-56,58,59], and 2 (18%) did so for the upper limb [51,53]. The main characteristics of these studies are listed in Table 1.

**Table 1:** Main characteristics of the study interventions.

Study	Sample	Age (years), mean (SD)	Study population	Intervention	Frequency of treatment (times/week)	Session time (minutes)	Total time of the intervention	Measurement instrument	Outcome	Results
Colomer et al [51]	30	58.3 (10.1)	Stroke	IG^a^: reverse study—A-B-A; A: conventional physical therapy program; B: AR^b^	3-5	45	12 weeks	Wolf motor function test, box and block test, 9-hole plug test, and Intrinsic Motivation Inventory	Elbow flexion and extension, wrist flexion and extension, finger flexion and extension, and grabbing different objects	Significant improvement in arm function and finger dexterity High levels of interest, motivation, and enjoyment
Lee et al [52]	21; CG^c^: 11; IG: 11	Not specified	Stroke	CG: general physical therapy program; IG: general physical therapy program+AR-based postural control program	CG: 5; IG: 3	30	CG: 4 weeks; IG: 8 weeks	TUG^d^, BBS^e^, spatial-temporal parameters (GAITRite), and dynamometer	Gait, balance, and muscle strength	Improvements in walking speed, balance and cadence, stride length, and stride length of paretic and nonparetic sides
Jung et al [50]	10; CG: 5; IG: 15	CG: 57.80 (10.23); IG: 58.40 (8.26)	Stroke	CG: FES^f^; IG: AR-based FES	3	20	4 weeks	Surface EMG^g^ machine, electronic goniometer, and manual muscle tester	Muscle activation, ankle range of motion, and muscle strength	Improved muscle activation in GCM^h^and TSA^i^ Improved muscle strength in dorsiflexion and plantar flexion
Kim et al [59]	28; group 1: 9; group 2: 10; group 3: 9	Group 1: 47.4 (8.4); group 2: 51.5 (12.9); group 3: 49.1 (11)	Stroke	Group 1: treadmill walking with EFS^j^ and AR therapy; group 2: treadmill walking with EFS therapy; group 3: gait on treadmill walking	3	20	8 weeks	BBS and TUG	Muscle strength, balance, and gait	Muscle strength increased significantly in groups 1 and 2. Balance and gait showed significant improvements in all groups.
Rothgangel et al [49]	75; group 1: 25; group 2: 26; CG: 24	61.1 (14.2)	Lower limb amputation	Group 1: mirror therapy+ AR teleprocessing; group 2: mirror therapy+self-administered mirror therapy; CG: sensory-motor exercises	Not specified	30	Group 1: 10 weeks; group 2: 10 weeks; CG: 10 weeks	NRS^k^ inventory of neuropathic pain symptoms, Patient-specific Functional Scale, EuroQol 5 Dimensions, Overall Perceived Effect Scale, and pain Self-Efficacy Questionnaire	Intensity, frequency, and duration of phantom pain	AR had no additional effects compared with the other groups.
Ortiz-Catalán et al [53]	14	50.3 (13.9)	Upper limb amputation	IG: motor execution in AR, game series; use of a virtual member in different tasks	2	120	12 sessions	NRS pain rating index, Weighted Scale of Pain Distribution, and study-specific frequency scale for each session	Intensity, frequency, duration, and quality of phantom limb pain (upper)	Clinical and statistical improvements in all phantom limb pain metrics
Lee et al [55]	30; group 1: 10; group 2: 10; group 3: 10	Women; group 1: 72.6 (2.67); group 2: 75.8 (5.47); group 3: 76.4 (5.54)	Older adults	Group 1: AR+Otago^l^; group 2: yoga; group 3: exercises at home	3	60	12 weeks	Strength of knee flexor, extensor, and ankle flexor muscles; footprint; static and dynamic load distribution; and MFS^m^	Muscle strength, balance, and risk of falling	Group 1, group 2, and group 3 had improved strength. The AR group improved significantly in balance and in the fall scale.
Yoo et al [56]	21; group 1: 10; group 2: 11	Women; group 1: 72.9 (3.41); group 2: 75.6 (5.57)	Older adults	Group 1: AR +Otago exercises; group 2: Otago exercises	3	50	12 weeks	Gait parameters, BBS, and FES-I^n^	Gait functionality, balance, and risk of falling	Group 1 had significant differences in gait and balance parameters greater than group 2. Group 1 had significant differences in fall prevention.
Ku et al [54]	36; CG: 18; IG: 18	CG: 65 (4.77); IG: 64.7 (7.27)	Older adults	CG: physical fitness program; IG: training with 3D-AR system	3	30	4 weeks	BBS, TUG, FAC^o^, MBI^p^, Fugl-Meyer lower limb subscale, Fugl-Meyer motor coordination, Fugl-Meyer motor score, and balance (Tetrax posturography)	Lower limb balance and lower limb mobility	Improved stability index with interaction between BBS and TUG Improvement in fall risk Improvement of the posturographic index Improved weight distribution index
Jeon et al [57]	27; CG: 13; IG: 14	CG: 72.71 (3.64); IG: 72.77 (3.79)	Older adults	CG: no exercise; IG: AR-based exercise	3	30	12 weeks	Stadiometer, BIA^q^, hand dynamometer, SFT^r^, and ESE^s^	Muscle mass, muscle function, physical performance, and exercise self-efficacy	Improved ASM^t^, SMI^u^, gait speed, SFT in chair stand test, 2MST^v^, and self-efficacy
Janssen et al [58]	16	Median: 69	Parkinson disease	Experimental condition: series of 180° turns with AR visual cues displayed through a HoloLens; 2 control conditions: with auditory cues and without any cues	1 session	N/A^w^	1 session	PTF^x^, mean number, and duration of FOG^y^ episodes; maximum medial COM^z^ deviation, maximum head-pelvis separation, and time to maximum head-pelvis separation; cadence, peak angular velocity, stride time, coefficient of variation, step height, and turn time	FOG parameters, axial kinematics, scaling, and timing of turning	AR visual cues did not reduce the PTF (*P*=.73) or the number (*P*=.73) and duration (*P*=.78) of FOG episodes, the peak angular velocity (visual vs uncued, *P*=.03; visual vs auditory, *P*=.02) and step height, and they increased the step height coefficient of variation and time to maximum head-pelvis separation. All FOG parameters were higher with AR visual cues than with auditory cues (PTF, *P*=.01; number, *P*=.02; and duration, *P*=.007 of FOG episodes).

^a^IG: intervention group.

^b^AR: augmented reality.

^c^CG: control group.

^d^TUG: Timed Up and Go.

^e^BBS: Berg Balance Scale.

^f^FES: functional electric stimulation.

^g^EMG: electromyogram.

^h^GCM: medial and lateral gastrocnemius.

^i^TSA: tibialis anterior.

^j^EFS: electrical functional stimulation.

^k^NRS: Numerical Pain Rating Scale.

^l^Otago: Strength and Balance Training Program for Seniors.

^m^MFS: Morse Fall Scale.

^n^FES-I: Short Falls Efficacy Scale–International.

^o^FAC: functional ambulation category.

^p^MBI: Modified Barthel Index.

^q^BIA: Bioelectrical Impedance Analysis (Inbody 720, Biospace).

^r^SFT: senior fitness test.

^s^ESE: exercise self-efficacy.

^t^ASM: appendicular skeletal muscle mass.

^u^SMI: skeletal muscle index.

^v^2MST: 2-minute step test.

^w^N/A: not applicable.

^x^PTF: percentage time frozen.

^y^FOG: freezing of gait.

^z^COM: center of mass.

The AR systems used varied widely: projectors connected to computers with webcams where images were shown [51], virtual upper limbs [53], training videos [50,55], teletreatment using AR with tablets [49], projections with AR on treadmills [55,56,59] or on the ground [55,56], a head-mounted AR device used for holographic display of AR visual cues [58], and an AR-based exercise rehabilitation system [57] or a newer system such as the 3D-AR system, in which the participant’s body movement was tracked, creating an AR environment that generated real images captured in videos with virtual images [54].

The intervention time ranged from 20 minutes [59] to 2 hours [53], although the most repeated chosen time was 30 minutes [49,52,54]. The most used frequency in the studies was 3 times per week [51,52,54,55,59] and 12 sessions [52-55].

The most widely used measurement scale in the selected studies was the BBS, used both for stroke [52,59] and in older adults [54,55]. Scales were also used to assess falls; in 1 trial, the Short Falls Efficacy Scale–International was used [56], and the Morse Fall Scale was used in another [55]. In both investigations, the target population was older adults. Another scale repeated in 3 of the selected articles was the TUG [52,54,59], which was applied to people with stroke and older adults. Regarding the studied population with amputations, many scales were used to assess pain; however, the only one in which both studies coincided was the Numerical Pain Rating Scale [49,53].

The results found regarding the interventions conducted in the field of physiotherapy were diverse in the different plots. In stroke, intensive and repetitive task-oriented exercises were used for upper limb functionality [51], postural control exercises [52], functional electrical stimulation [50], and treadmill [59]. Mirror therapy and sensorimotor exercises were used in the treatment of phantom limb pain [49]. In Parkinson disease, turns around the patient’s axis were used [58]. Finally, in geriatrics, exercises from the Otago protocol were used [55,56], as well as yoga [55] and physical conditioning with strengthening and balance training [54,57].

### Methodological Quality of the Included Studies

The results of the methodological quality assessment can be found in Table 2. After assessing the studies using the PEDro scale, it stands out that, of the 11 studies included in the review, 7 (64%) had high methodological quality (≥6 points), and the rest were acceptable. The scores obtained and the detailed characteristics of each study are shown in Table 2. Regarding the Scottish Intercollegiate Guidelines Network scale, most studies had a grade of B (Table 3).

**Table 2:** Evaluation of the methodological quality according to the PEDro scale^a^.

Study	Item 1^b^	Item 2^c^	Item 3^d^	Item 4^e^	Item 5^f^	Item 6^g^	Item 7^h^	Item 8^i^	Item 9^j^	Item 10^k^	Item 11^l^	Score (out of 10)
Colomer et al [51]	1	0	0	1	0	0	0	1	1	0	1	4
Lee et al [52]	0	1	0	1	0	0	1	1	1	1	1	7
Jung et al [50]	1	1	1	1	0	0	0	1	0	1	0	6
Kim et al [59]	1	1	0	1	0	0	0	0	0	1	1	4
Rothgangel et al [49]	1	1	1	1	0	0	1	1	1	1	1	8
Ortiz-Catalán et al [53]	1	0	0	1	1	1	1	1	1	0	1	7
Lee et al [55]	1	1	0	1	0	0	0	0	0	1	1	4
Yoo et al [56]	0	1	0	1	0	0	0	1	0	1	1	5
Ku et al [54]	1	1	1	1	0	0	1	1	0	1	1	7
Jeon et al [57]	1	1	1	1	0	0	0	1	0	1	1	6
Janssen et al [58]	1	0	1	1	0	0	1	1	0	1	1	7

^a^1=yes and 0=no.

^b^Choice criteria specified; did not add up in the final computation.

^c^Random assignment.

^d^Covert assignment.

^e^Baseline similarity.

^f^Subject blinding.

^g^Therapist blinding.

^h^Evaluator blinding.

^i^Greater than 85% follow-up for at least 1 key outcome.

^j^Intention-to-treat analysis.

^k^Statistical comparison between groups for at least 1 key outcome.

^l^Point measures and variability for at least 1 key outcome.

**Table 3:** Grades of recommendation according to the Scottish Intercollegiate Guidelines Network scale.

Study	Grade of recommendation
Colomer et al [51]	B
Lee et al [52]	A
Jung et al [50]	B
Kim et al [59]	B
Rothgangel et al [49]	A
Ortiz-Catalán et al [53]	B
Lee et al [55]	B
Yoo et al [56]	B
Ku et al [54]	A
Jeon et al [57]	B
Janssen et al [58]	B

### Risk of Bias

The results of the risk of bias can be observed in Table 4. It should be noted that 36% (4/11) of articles presented a low risk of selection bias, as they were randomized [49,52-54], although only 25% (1/4) of them also presented allocation concealment [54]. With respect to performance bias, none were at low risk. Regarding detection bias, 45% (5/11) of the articles included in the review were at low risk. In relation to dissertation bias, all of them were at low risk.

**Table 4:** Risk of bias.

Author	Criteria (risk)						
	1^a^	2^b^	3^c^	4^d^	5^e^	6^f^	7^g^
Colomer et al [51]	High	High	High	High	Low	Unclear	Unclear
Lee et al [52]	Low	High	High	Low	Low	Unclear	Unclear
Jung et al [50]	Low	Unclear	High	High	Low	Unclear	Unclear
Kim et al [59]	Unclear	High	High	High	Low	Unclear	Unclear
Rothgangel et al [49]	Low	High	High	Low	Low	Unclear	Unclear
Ortiz-Catalán et al [53]	Low	High	High	Low	Low	Unclear	Unclear
Lee et al [55]	Unclear	High	High	Unclear	Low	Unclear	Unclear
Yoo et al [56]	Unclear	High	High	Low	Low	Unclear	Unclear
Ku et al [54]	Low	Low	High	Low	Low	Unclear	Unclear
Jeon et al [57]	Low	Unclear	High	High	Low	Unclear	Unclear
Janssen et al [58]	High	High	High	High	Low	Unclear	Unclear

^a^Random sequence generation (selection bias).

^b^Allocation concealment (selection bias).

^c^Blinding of participants and personnel (performance bias).

^d^Blinding of outcome assessment (detection bias).

^e^Incomplete outcome data (attrition bias).

^f^Selective reporting (reporting bias).

^g^Other bias.

### Study Groups Included in the Meta-analysis

**Figure 2:**
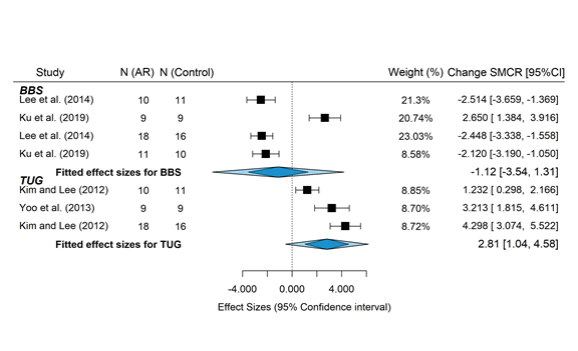
Forest plot. AR: augmented reality; SMCR: standardized mean change with raw standardization; BBS: Berg Balance Scale; TUG: Timed Up and Go.

**Figure 3:**
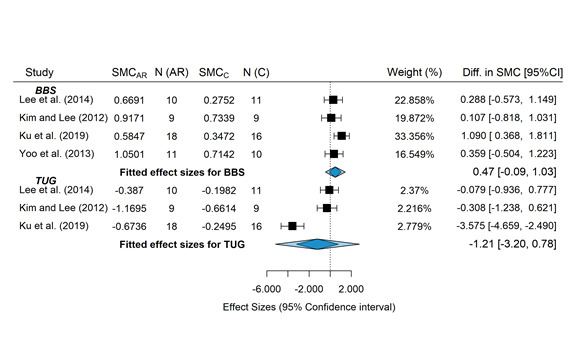
Weights and sample size of each study. SMC: standardized mean change; AR: augmented reality; BBS: Berg Balance Scale; TUG: Timed Up and Go.

**Figure 4:**
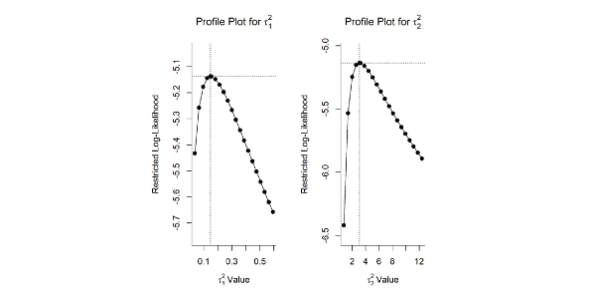
Variance components.

**Figure 5:**
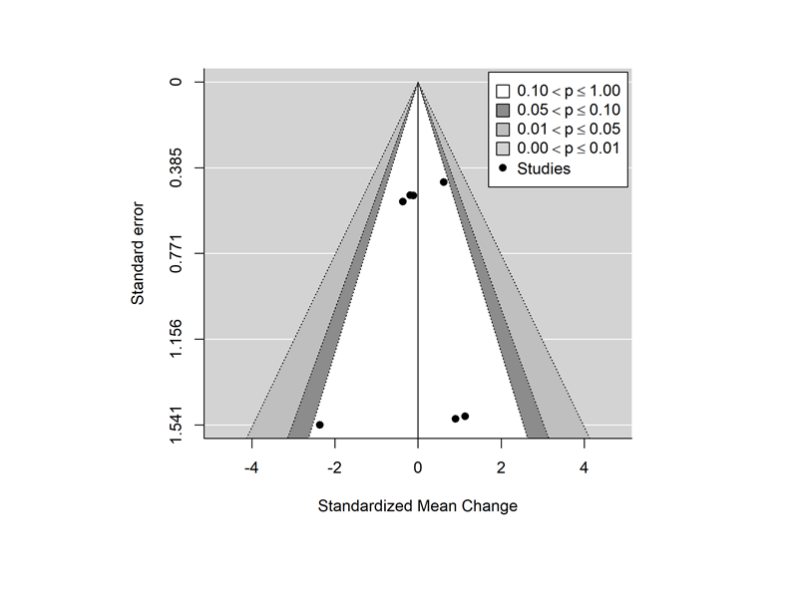
Funnel plot of the standardized mean change versus the standard error.

In this meta-analysis, 36% (4/11) of studies were selected to evaluate the differences in mean changes in BBS and TUG scores. The power for detecting differences was low because of the reduced number of studies and small sample sizes. The data used for the meta-analysis are shown in Table 5. Descriptive data extracted from the selected studies are included in Multimedia Appendix 1 [49,51-56,59]. The (pooled) difference in standardized mean change was 0.473 (95% CI −0.0877 to 1.0338; *z*=1.65; *P*=.10) for the BBS and −1.211 (95% CI −3.2005 to 0.7768; *z*=−1.194; *P*=.23) for the TUG, both differences favoring the intervention group, although the null hypothesis cannot be rejected. The forest plot (Figure 2 [49-59]) showing the individual and pooled SMCR (with 95% CI), weights, and sample sizes of each study is shown in Figure 3 [49-59]. Substantial heterogeneity (test for residual heterogeneity: *Q*_5_=45.82; *P*<.001) was present among the studies, with estimated variance components of 0.148 (95% CI 0.0001-2.2727; τ2BBS) and 3.098 (95% CI 0.5818-36.1115; τ2TUG). No identifiability problems for the variance components were found (Figure 4). The 2 outcomes showed a very high correlation (ρ=−0.99). The individual effect size was significant for both outcomes in the study by Ku et al [54], the study with the greater sample size. Nonetheless, sensitivity analysis showed that this study had higher standardized residuals and Cook distance values for the outcome TUG. The contour-enhanced funnel plot (Figure 5) seems to indicate the absence of publication bias (results should be considered with caution because of the small sample size).

**Table 5:** Data used for the meta-analysis.

Study and outcome			SMC^a^				Correlations between pre- and postintervention means					SD prediction interval			Sample sizes					Pooled correlations between the 2 outcomes			Differences in SMC			Sampling variance			Sampling covariance
			Control		Intervention		Control		Intervention						Control		Intervention												
**Lee et al [52]**																													
	BBS^b^	0.2752		0.6691		0.5347075		0.8318219		1.3679			10			11		0.6482			0.2879			0.1929			0.1235		
	TUG^c^	−0.1982		−0.3870		0.4264936		0.7023536		2.3817			10			11		0.6482			−0.0792			0.1911			0.1235		
**Kim and Lee [59]**																													
	BBS	1.3922		0.9171		0.9558409		0.8978624		1.7163			9			9		0.6482			−0.2768			0.2244			0.1452		
	TUG	−0.5768		−1.1695		0.7852951		0.9161890		1.6490			9			9		0.6482			−0.3594			0.2258			0.1452		
**Ku et al [54]**																													
	BBS	0.3472		0.5847		0.7299132		0.9078490		0.2180			18			16		0.7069			1.0895			0.1355			0.0548		
	TUG	−0.2495		−0.6736		0.5644717		0.9050366		0.1187			18			16		0.7069			−3.5745			0.3060			0.0548		
**Yoo et al [56]**																													
	BBS	0.7142		1.0501		0.8919225		0.9560710		0.9351			11			10		0.7069			0.3593			0.1940			0.1940		

^a^SMC: standardized mean test.

^b^BBS: Berg Balance Scale.

^c^TUG: Timed Up and Go.

## Discussion

### Principal Findings

In this systematic review and meta-analysis of clinical trials, we wanted to determine the use of AR in conjunction with conventional therapy in the different fields of physiotherapy. In our study, favorable results were obtained in balance and gait [54,59], upper limb functionality [51], muscle mass, physical performance, and exercise self-efficacy [57] and in reducing the risk of falls [54-56] and pain in phantom pain syndrome [53]. In addition, significant differences were found with respect to conventional therapy. This intervention was implemented for stroke, amputations, older adults, and Parkinson disease [49,51-56,59]. These findings are consistent with those of other studies in healthy participants, such as the study by Bennour et al [28], which showed promising results for retraining the lower limb in gait through footprint modifications using AR, or the upper limb in the trial by Cavalcanti et al [30] using the AR device *ARkanoidAR*, which improved and corrected movement with the use of auditory, textual, or imaging feedback.

The aspects related to AR interventions and their positive results are as follows. Regarding their use in patients with amputations, the 2 articles found conflicting results on AR in phantom limb pain. In the trial by Ortiz-Catalán et al [53], pain was significantly reduced using AR; however, Rothgangel et al [49] found no additional effect compared with the other groups. As a possible cause, Rothgangel et al [49] argued that an inconsistency during teleprocessing with the representation of the amputated limb could have led to a lack of integration.

In patients who had a stroke, we found improvement in the functionality of the upper limb [51], with high motivation among participants and improvements in the strength of the lower limb, balance, and gait. Protocol studies on these last 2 variables have also been found in stroke, with the AR therapy C-Mill [60] and the Gait Adaptation for Stroke Patients with AR system [61], which have not yet yielded results. AR also appears promising for the rehabilitation of hand-eye coordination and finger dexterity [62].

Regarding geriatrics, favorable results were found in lower limb strength, balance, muscle mass, physical performance, exercise self-efficacy, and fall prevention. It is in this area that we have seen greater consistency in the findings. In this sense, for older adults who normally depend on visual information to achieve balance, AR training could effectively improve proprioception of the lower limbs, favoring static balance. It would be even better if the used system provides visual feedback [54].

There are other areas within physiotherapy where AR could be used to improve these parameters, such as Parkinson disease, where VR has been used to improve balance [63]. Experiments are also being conducted with a platform based on AR and the Microsoft Kinect v1 sensor, where various exercises are implemented with linear or circular movement patterns that allow the physiotherapist to adjust them to the patient’s abilities, although there are still no results [64]. However, AR visual cues did not improve freezing of gait, impaired axial kinematics, or turn scaling and timing [58].

It was possible to conduct the meta-analysis by taking into account the BBS and TUG. The BBS comprises 14 items where the patient is asked to perform several specific tasks to check their balance. Total scores range from 0 (severely affected balance) to 56 (excellent balance) [65]. Individuals with values ≤45 are at greater risk of falling [66]. With respect to the TUG, it is a scale that serves to check a patient’s balance and risk of falling [67]. A duration of ≥13.5 seconds on the TUG is associated with a greater risk of falling in older adults and in people with vestibular dysfunction [68]. With the results obtained in both subgroups—BBS [52,54,56,59] and TUG [52,54,59]—the global result of the meta-analysis was favorable so that the intervention using AR is effective for the improvement of balance. However, given the small size of the samples, the heterogeneity of the populations studied, measuring instruments, methods used, times of application, and frequency and duration of the treatments, the results were not conclusive.

Advantageous aspects of AR use have also been described [51-56,59]. However, the procedures used were different in each study [49,51-56,59]. This may lead to uncertainty in the choice of a system for AR and physiotherapy development. Regarding the systems used, although in the past decade they were much more complex [36], they should be simpler in the future. With the present advances in AR systems, such as the HoloLens, its application in clinical settings could be expected to increase [69].

Displays used in AR can be classified into the following categories: head-worn, handheld, and projective [36]. In our research, most of them were projective, except in 2 of the studies [50,58], where head-mounted devices were used. Regarding the classification of the AR system by levels [70], all the systems used in our review were level 3, in which AR is displayed on screens and transformed into augmented vision through projectors that allow the real environment to become an immersive virtual world. The exception was the study by Ortiz-Catalán et al [53], in which a level 1 was used through markers, from which the 3D information contained was extracted, showing it through a device screen. In relation to the type of feedback used, our findings were the same as those of Hussain Al-Issa [36], where it was of a visual type, although in one of the studies in our paper, there was also auditory feedback [59].

There are some obstacles that limit the generalized use of AR, such as technological and user interface limitations [71]. Other negative aspects such as eye fatigue or human factors related to the effects of long-term use, such as latency and the user’s adaptation to the equipment, could also reduce task performance. In addition, depth perception can make objects appear farther away than they really are [72]. It also seems that AR has not been used because, for the same objective, other technologies with easier approaches could be used, such as VR [10,69].

However, it should be considered that AR has certain advantages that VR does not have. For example, VR cannot recognize the real dangers that can cause injury, whereas, in AR, the patient is aware of the possible risks [73]. In addition, the participant can interact with the application, the environment, and tangible objects [36], as AR has greater proprioceptive feedback [74]. Game-based rehabilitation would also be of interest to create an interface (means) suitable for AR, encouraging the use of personalized games, which could improve motivation by taking into account whether the game is meaningful and motivating, the type of feedback obtained, the usability, and the interaction technique used with the environment [69].

Furthermore, the benefits of AR in the use of telerehabilitation demonstrate its effectiveness in the remote monitoring of the patient and can even modify according to their progress, providing high-quality attention with reduced costs [75,76]. Thus, the development of an AR system on mobile devices could be a good alternative for patients [77]. It seems that physiotherapy has not yet discovered all the potential promised by AR. What does appear to have been a common approach to the use of AR is lower limb recovery for fall prevention and improved balance.

This study may serve as an aid in clinical practice through the use of AR systems. It may also serve as a preliminary step toward further research with a more homogeneous methodology and the ability to experiment with these technological systems in other areas of physiotherapy where pain, functionality, balance, and fall prevention may be an objective to be pursued.

### Limitations

In terms of the limitations found, we must mention the limited number of studies with low quality and the wide variety of AR interventions with respect to the system used, number of sessions, and frequency and duration of the treatment sessions. There was no homogeneity with respect to the instruments used to measure the variables studied or the variables themselves. Similarly, the need for authors to use the same measuring instruments stands out as, in some cases, it was not possible to compare studies statistically because different versions of the same scale or different units of measurement were used.

### Comparison With Prior Work

After 10 years of the review by Hussain Al-Issa [36] and with results in promising pilot studies where a great future is always foreseen, our search shows the opposite. We found few studies with considerable heterogeneity and few physiotherapy plots. A difference found with respect to this previous review is that AR is now being used in telerehabilitation [8,34,76,78], although more research is needed.

### Conclusions

According to the results obtained, we can say that AR, in combination with conventional therapy, has been used for physical performance, treatment of balance and prevention of falls in geriatrics, functionality of the lower limb and upper limb in stroke, and pain in phantom pain syndrome. However, no positive results were obtained with turning and timing in the freezing of gait in Parkinson disease. Owing to the diversity of the interventions and the variables measured, no consensus can be reached on the best AR system in each area studied, although the most commonly used were the level 3 projectives.

Future clinical trials are needed using larger sample sizes and with greater homogeneity in terms of the devices used and the frequency and intensity of the interventions.

## Appendix

Multimedia Appendix 1 Descriptive data extracted from the selected studies.

## Abbreviations

AR: augmented reality
BBS: Berg Balance Scale
PRISMA: Preferred Reporting Items for Systematic Reviews and Meta‐Analyses
PROSPERO: International Prospective Register of Systematic Reviews
SMCR: standardized mean change with raw standardization
TUG: Timed Up and Go
VR: virtual reality
